# Dispensers and the Insurance Bill

**Published:** 1911-10-28

**Authors:** 

**Affiliations:** Apothecaries' Hall, London


### The Editor's Letter Box.

### DISPENSERS AND THE INSURANCE BILL.

A Certificated Dispenser, Apothecaries' Hall, London, writes:—The National Insurance Bill is an exceedingly far-reaching measure, and it is impossible at present to see how far its embraces will extend. Those in the best position to form an opinion as to its effect upon the practice of the pharmaceutical chemists, using the term in its broadest sense, and including all those whose business is that of dispensing medicines, presage a revolution. With the medical requirements in the form of medicines, drugs, and appliances of 15,000,000 of people suddenly switched off from the doctors on to the chemists, it cannot be otherwise. The only source of supply for insured persons is to be in future through persons or firms entitled to carry on the business of a chemist, in selling and dispensing poisons in accordance with the Pharmacy Acts. Hitherto the only legal dispensing qualification has been the Assistant Apothecaries' certificate granted after examination, by the Society of Apothecaries of London, under the provisions of the Apothecaries Act, 1815. It is unnecessary here to refer to the ancient origin or early history of this Society. A Latin inscription upon one of the walls of their Hall in Water Lane records that upon this site the Apothecaries Pharmaceuticæ Societatis Londinensis had its hall, which was burnt in the great fire, and the present edifice was erected a few years after. In 1813 a Bill was introduced into the House of Commons "for regulating the practice of Apothecaries, Surgeons, Practitioners in Midwifery, and Compounders and Dispensers of medicine." This Bill, among other things, provided that no qualified compounder should enter the service of an unqualified compounder; the Bill, however, was not proceeded with.

### Some Ancient History.

But in 1815 the Apothecaries Act was enacted. This was a public Act to regulate the practice of Apothecaries in England and Wales, and is still on the statute book. A clause of this Act empowers the Society of Apothecaries to examine persons as to their fitness and qualification to act as assistants to apothecaries, and to grant certificates to persons who satisfy the examiners. The regulations for the examination of Apothecaries' Assistants, as published in *The Gentleman's Magazine* for December, 1815, were as follows:—"That every person who shall be admitted to an examination for a certificate to act as an assistant to any Apothecary, in compounding and dispensing medicines, shall be required to translate parts of the 'Pharmacopiæ Londinensis,' and physicians' prescriptions, and shall be examined as to his knowledge of pharmacy and materia medica." The Society have from time to time added to and improved the requirements of the examination, so that now, in the words of the *B.M.J.*, "the examination is an eminently practical one." Notwithstanding the Act which ratified their ancient rights, the apothecaries could not restrain unqualified persons from calling themselves apothecaries; many did so, others put over their doors the words "chemist and druggist," or "pharmaceutical chemist." The Society of Apothecaries were powerless to take action unless they could prove "practising"—viz. visiting, diagnosing, and prescribing for payment. This state of things led to the co-operation of the Colleges of Physicians and Surgeons with the Apothecaries, who proposed legislation by which the chemists and druggists were to be placed under the government of these corporations, chiefly under the Apothecaries.

### The Pharmaceutical Society Incorporated.

To avert this danger the druggists formed themselves into a society, and in 1843 they were incorporated as the Pharmaceutical Society, and in 1852 an Act of Parliament giving them power to examine and certify pharmaceutical chemists and assistants to pharmaceutical chemists. Further powers were obtained in 1868 in an Act to regulate the Sale of Poisons and amend the Pharmacy Act, 1852. Neither of these Acts mentions the Apothecaries' Assistants' qualification nor interferes with dispensing physicians' prescriptions; because (*Lancet*, July 11, 1868) "the simple principle of the Bill is to provide that those who sell poisons shall be properly educated and in some degree responsible, there is not a clause which means anything else." The National Health Insurance Bill strikes a new note, deals with the dispensing of prescriptions, creates a class of State dispensers, and *entirely ignores legally qualified persons* who have been certified by a competent authority acting under statutory powers.

### Prescribing and Dispensing.

The great question of the separation of prescribing from dispensing has been considered by both doctors and chemists for years. Both have longed for the day when such a state could be possible. Few, if any, medical men dispense because they like it, but because they are compelled by present circumstances to do it. The remarks which have been made in certain quarters *re* the evils of doctors dispensing would perhaps have been better left unsaid. I will not say anything about glass-houses and stones, but I am old enough to remember the days when quinine was dear and Food and Drugs Acts were still dreams, and have "under the superintendence of a registered pharmacist" prepared quinine-wine, the bitterness of which was certainly not due to quinine. I have also dispensed for many medical men, and I have never known one who, solely because his profit would be less, has prescribed for his patient anything other than what he has considered best suited for the case. Cost is not the sole criterion of efficiency. Mist. alb. is not expensive, yet even if it is called "hospital whitewash," it has been known to do good. Calomel is also cheap and effective. A little money so spent in Ferri et ammon. cit. will provide a lot of useful medicine. Are these to be tabooed simply because they are cheap? Lotio nigra is not dear, but for its purpose is as good as if costing a guinea a bottle. Hydrarg. cum cretâ, again, costs less than 606. Are State patients to demand the latter simply because it costs more?

### The Patients at Cheap Dispensaries.

Respecting the cheap "dispensing doctors who give a week's attendance and two bottles of medicine for a shilling," I, without attempting to justify such practice, have no doubt they fill a want; but they should not be necessary. Will the Bill remove the need? Who are the people who crowd these places? They are mostly poor, many of them home workers, others able to do casual work, in many cases on the border-line of destitution. They are unable to attend at the out-patients' department of the hospitals, where in many cases a visit means hours; they are too poor to pay the usual fees, perhaps not destitute enough to come under the Poor Law, or perhaps too independent to ask for a parish order, or too ignorant to know that the receipt of medical relief neither pauperises nor disqualifies for an old-age pension. To remedy this, make the Poor-Law dispensaries more accessible, open them in the evenings if necessary, thus those may be helped whom the Chancellor would like to help, but who on account of cost he is obliged to leave out of the Bill.

Who Shall Dispense

But to return to dispensing, most are agreed that it will be best to separate prescribing from dispensing, but who shall dispense the people's medicine? Shall the monopoly be in the hands of those registered by the Pharmaceutical Society, or shall the certificated dispensers of Apothecaries' Hall, as is their due, be accorded an equal right? The examiners appointed under the provisions of the Apothecaries Act are gentlemen of the highest attainments, and the Apothecaries' Assistants' diploma is recognised by the Local Government Board for dispensers in institutions under its control, as well as by a great number of other institution committees, and it is held by hundreds of men and women who fill responsible positions as dispensers in hospitals, infirmaries, asylums, and dispensaries. Nevertheless the House were assured in the course of the debate on Clause 14 that holders of this qualification could only act when under the supervision of a qualified chemist. This would be amusing were it not a matter of such serious importance to the certificated dispensers, seeing that for half a century after their examination was instituted the qualified chemist was unknown. Can this Bill, which so vitally affects them, ignore them when "our lawyers with justice tell us that the law is the perfection of wisdom, and what is reason is not law?"

